# Similar folds with different stabilization mechanisms: the cases of prion and doppel proteins

**DOI:** 10.1186/1472-6807-6-17

**Published:** 2006-07-21

**Authors:** Stefano Colacino, Guido Tiana, Giorgio Colombo

**Affiliations:** 1 Istituto di Chimica del Riconoscimento Molecolare, CNR, Via Mario Bianco 9, 10131 Milano, Italy; 2 Dipartimento di Fisica, Università di Milano and INFN, Via Celoria 16, 20133 Milano, Italy

## Abstract

**Background:**

Protein misfolding is the main cause of a group of fatal neurodegenerative diseases in humans and animals. In particular, in Prion-related diseases the normal cellular form of the Prion Protein PrP (*PrP*^*C*^) is converted into the infectious *PrP*^*Sc *^through a conformational process during which it acquires a high β-sheet content. Doppel is a protein that shares a similar native fold, but lacks the scrapie isoform. Understanding the molecular determinants of these different behaviours is important both for biomedical and biophysical research.

**Results:**

In this paper, the dynamical and energetic properties of the two proteins in solution is comparatively analyzed by means of long time scale explicit solvent, all-atom molecular dynamics in different temperature conditions. The trajectories are analyzed by means of a recently introduced energy decomposition approach (Tiana et al, *Prot. Sci*. **2004**) aimed at identifying the key residues for the stabilization and folding of the protein. Our analysis shows that Prion and Doppel have two different cores stabilizing the native state and that the relative contribution of the nucleus to the global stability of the protein for Doppel is sensitively higher than for PrP. Moreover, under misfolding conditions the Doppel core is conserved, while the energy stabilization network of PrP is disrupted.

**Conclusion:**

These observations suggest that different sequences can share similar native topology with different stabilizing interactions and that the sequences of the Prion and Doppel proteins may have diverged under different evolutionary constraints resulting in different folding and stabilization mechanisms.

## Background

The molecular determinants of neurodegenerative diseases have been the subject of very intense research over recent years [1,2]. Particular attention in this field has been devoted to Prion proteins (*PrP*) due to their fundamental role as infective agents in diseases generally known as Transmissible Spongiform Encephalopathies (TSE) that affect humans and animals, including Creuzfeld-Jakob disease, fatal familial insomnia and Gerstmann-Straussler-Scheinker disease in humans, scrapie in sheep and mad-cow disease in cattle. The distinctive trait of Prion-related diseases is that *PrP *proteins seem to act as the only infectious agents, with no intervention of genetic material, by causing self-propagating conformational changes [3-5]. Experimental evidences unveiled that in all these cases the normal and benign form of the Prion Protein (*PrP*^*C*^) can undergo a conformational change of the native state leading to a new isoform designated *PrP*^*Sc *^which is insoluble, characterized by an increased content in *β*-structure and with a high tendency to form amyloid aggregates [5,6]. Misfolding to the pathological species occurs through the unfolding of the α-helical-rich conformation and refolding to a β-sheet rich one [6,7]. Once formed, *PrP*^*Sc *^can interact with other monomeric PrPs, acting as a template to speed up the conversion of the normal form to the scrapie one. Interestingly, more than 20 mutations distributed throughout the sequence of PrP have been shown to lead to neurological disorders: their role has been suggested to be connected with either the lowering of the free-energy barrier in the conformational conversion favoring the formation of *PrP*^*Sc*^, or with an increase in the oligomerization rate of the insoluble isoforms [8].

**Figure 1:**
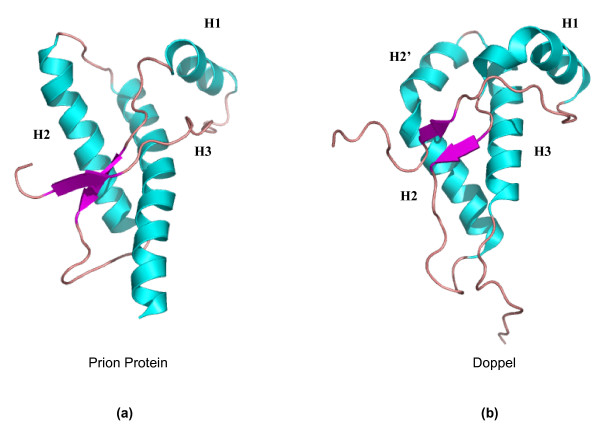
**Starting 3D structures of Prion and Doppel**. PDB derived starting structures of the Prion Protein (a) and of the Doppel Protein (b). The two structures are coloured according to secondary structures. Different secondary structure elements are also labelled in bold characters.

Despite the vast amount of research carried out on the Prion Protein, its physiological function is still unknown. Recent results have shown that the N-terminal unstructured region of the protein binds Cu(II) ions suggesting that it should be implicated in copper transport and regulation [9,10]. Other studies suggested that *PrP*^*C *^may function in signal transduction through a pathway involving Fyn Kinase [11]. In this context, it was hoped that the study of mice animal models in which the *Prnp *gene was deleted could provide clues on the function of PrP. Deletion of the *Prnp *gene actually eliminated susceptibility to prion infection with *PrP*^*Sc*^. However, this caused the degeneration of Purkinje neurons, causing a different type of neurological disease [12]. The cause for this behavior was associated with a paralog of the *Prnp *gene, termed *Prnd*, with about 25% sequence identity to the PrP gene. In particular, *Prnd *encoded for a different protein named Doppel (*Dpl*) which has a 3D structure and native topology almost identical to *PrP*^*C*^, in spite of the low sequence similarity (25%) (Figure 1) [13]. Most interestingly, *Dpl *does not convert to a different conformation, i.e. it seems to cause neurodegeneration without a transition to an analogous of the Scrapie form of Prion Protein [13].

These observations suggest that, despite the structural similarities, there may be fundamental differences in the stabilization and unfolding/misfolding mechanisms of *PrP *and *Dpl*, which may be strictly connected to the interactions among residues in the native state.

In this paper we make use of long-timescale, explicit-solvent, all-atom simulations of the structured part of the human PrP protein (residues 125 to 229, pdb code 1qlz) [14], and of the Doppel protein (*Dpl*, pdb code 1i17) [13] (see Fig. 1a and 1b). These simulations are used as a basis to perform an analysis of the stabilization energy of the two proteins, in order to obtain direct information on the determinants of their (de)stabilization and indirect information on the associated folding properties. MD simulations for both proteins were thus run at 310 K for 50 ns with protonation conditions of the titratable groups consistent with pH 7 (see the Methods section). The final structures of each simulation at room temperature were used as starting points for two more simulations at 350 K for 20 ns at pH 7, and after this time span the temperature was raised to 450 K for 20 more nanoseconds to speed up the complete unfolding of the proteins and to investigate possible pathways to the formation of infectious species.

The main goal is to shed light on the different role that structural motifs and specific residues play in the (de)stabilization of native structures of the two proteins. In particular, we have used a simple energy-analysis approach developed in our group to obtain a detailed picture of the sites mostly responsible for the stability of the native state of each protein in selected environments [15]. The energy analysis is based on an eigenvalue-decomposition of the symmetric interaction energy matrix obtained by the calculation of all the interactions between non consecutive residues along an MD trajectory. The analysis of the components of the eigenvector associated with the lowest eigenvalue has proven useful to identify those sites mostly responsible for protein stabilization in a series of uncorrelated test proteins and in a family of proteins sharing the same 3D fold with low sequence identity [15-17]. One can thus investigate and highlight the main differences in the dynamics and in the energy distribution in the native states of *PrP *and *Dpl*, and correlate them with the impact that topological and sequence differences may ultimately have on the presence or absence of the structural rearrangements which are at the basis of neurodegenerative diseases.

## Results and discussion

The structural properties of *PrP *were already discussed elsewhere [16], so that in this paper we will concentrate on the structural properties of *Dpl *and on their comparison with those of *PrP *at pH 7.

**Figure 2:**
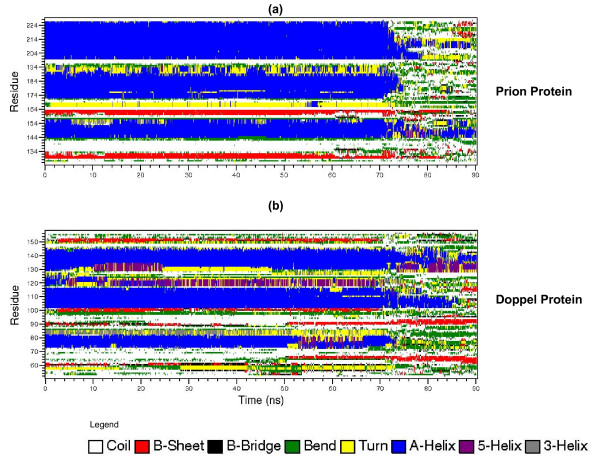
**Secondary Structure Evolution**. Time evolution of the secondary structures calculated according to the DSSP algorithm of *PrP *(a) and of *Dpl *(b) during the simulations. Between 0–50 ns the temperature was 310 K; 50–70 ns 350 K; 70–90 ns 450 K.

**Figure 3:**
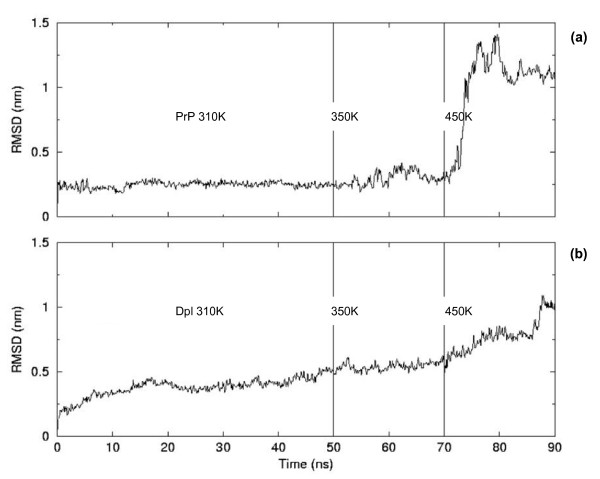
**Time evolution of the RMSD from the Native Structure**. Time evolution of the Root Mean Square Deviation (RMSD) of the structures sampled from the trajectories from the native structure of *PrP *(a) and of *Dpl *(b) during the simulations. Between 0–50 ns the temperature was 310 K; 50–70 ns 350 K; 70–90 ns 450 K.

**Figure 4:**
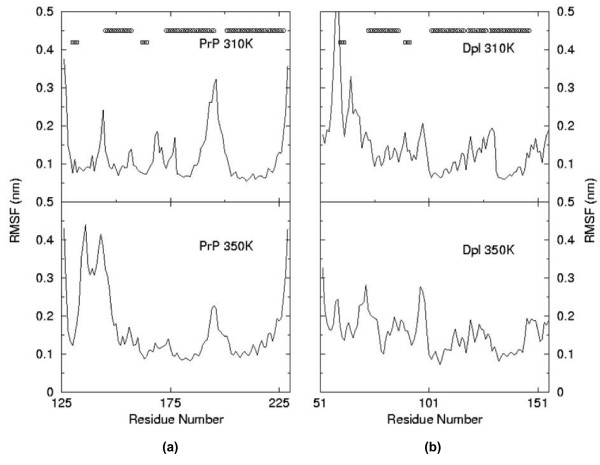
**Structural Flexibility of *PrP *and *Dpl***. (a) Flexibility of the *PrP *at 310 K, top panel and at 350 K bottom panel. (b) Flexibility of the *Dpl *at 310 K, top panel and at 350 K bottom panel.

Both proteins tend to conserve their overall tertiary and secondary structural arrangements at both 310 K (first 50 ns of the simulations) and 350 K (interval between 50 and 70 ns of the simulations) (Fig. 2a and 2b), indicating the absence of major conformational changes or sub-global unfolding processes at these temperatures, as expected when considering that the Tm is about 60°C for PrP and about 50°C for Dpl, and that the characteristic time for unfolding is known to exceed 20 ns at 350 K. This is also evident in the two RMSD plots for the same simulations (Fig. 3a and 3b). Interestingly, the ordered secondary structure of the C-terminal part of helix H2' (after the helix kink) and the N-terminal part of helix H3 in *Dpl *appear to be unstable and undergo transitions to disordered conformations. The comparison of the flexibility properties of the two proteins, calculated as the Root Mean Square Fluctuation (RMSF) per residue over the whole simulation length, already highlights a difference between them. In the case of *PrP *at pH 7, a substantial increase in the flexibility can be noticed in the N-terminal part of the molecule when raising the temperature from 310 to 350 K. *PrP *helix H1 fluctuates as a rigid body conserving its secondary structure. In contrast, the flexibility properties of *Dpl *appear to be much less sensitive to variations in external conditions (Fig. 4), except for the disordered N-terminal region which displays high fluctuations at 310 K. However, in the last 5 ns of the 310 K simulation a stabilizing interaction between the N-terminal R51 and Q85 is established and not broken in the subsequent part of the simulation at 350 K. Interestingly, the regions encompassing the two β-strands and most of the H1 α-helix are of low flexibility at both temperature conditions. In contrast, higher values are observed for the loop connecting the second β-strand and the N-terminal part of helix H2, and for the terminal part of helix H2 after the kink (labeled H2'), despite the presence of a well defined secondary structure. These data are consistent with experimentally derived flexibility measures based on heteronuclear NOE determinations [13]. These first characterizations of the dynamical properties suggest that the two proteins, despite sharing the same global 3D topology, differ in their finely detailed flexibility properties and fluctuations around the native conformation.

To gain a deeper understanding of the molecular interactions responsible for the differential behavior of the two molecules, we analyzed directly the distribution of stabilization energy among the residues of the two proteins, as described in the Methods. In brief, it was shown that single domain proteins usually display a core of few residues stabilizing collectively the whole protein [15]. The lowest eigenvalue λ_1 _of the residue-residue interaction matrix obtained by the simulated trajectory is, for core-stabilized proteins, consistently lower than the other eigenvalues. The elements of the associated eigenvectors indicate to which extent each amino acid participates to the core (for details on the application of the method to small single-domain proteins see references [15-17]). The details of eigenvectors corresponding to higher eigenvalues are reported in the supplementary material [see Additional file 1].

The results of the energy analysis show quite substantial differences among the two proteins. First of all, the ratio of the separation between the first two eigenvalues Δλ_12 _and the average spacing between all the others Δλ¯
 MathType@MTEF@5@5@+=feaafiart1ev1aaatCvAUfKttLearuWrP9MDH5MBPbIqV92AaeXatLxBI9gBaebbnrfifHhDYfgasaacH8akY=wiFfYdH8Gipec8Eeeu0xXdbba9frFj0=OqFfea0dXdd9vqai=hGuQ8kuc9pgc9s8qqaq=dirpe0xb9q8qiLsFr0=vr0=vr0dc8meaabaqaciaacaGaaeqabaqabeGadaaakeaadaqdaaqaaiabfs5aejabeU7aSbaaaaa@2FD7@ is much higher for *Dpl *than for *PrP *in all conditions. This ratio quantifies to which extent the stabilization energy of the protein is concentrated in a few, mutually interacting residues and, consequently, to which extent the overall stabilization energy is well accounted for by the first eigenvector μ^1^. In particular, at pH 7 the Δλ_12_/Δλ ratio for the *Dpl *protein is 10.53 at 310 K and 9.17 at 350 K, while for the *PrP *these values decrease to 3.03 and 2.33 respectively.

**Figure 5:**
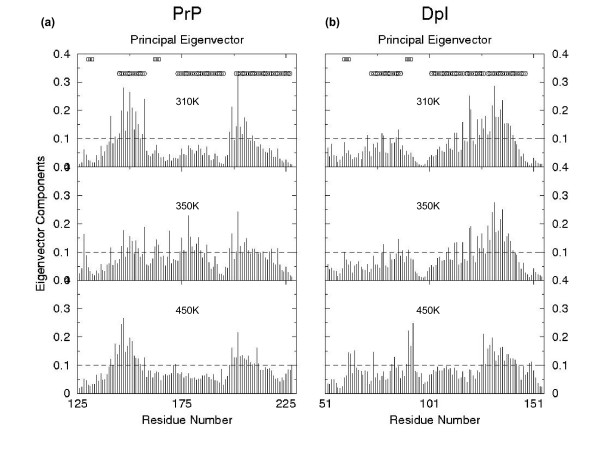
**Principal Eigenvector Components**. The components of the eigenvectors associated with the largest eigenvalue from the energy decomposition analysis in the all-atom MD simulations for (a; left panel) the *PrP *protein and (b; right panel) the *Dpl *protein at different temperatures, reported in each single panel. The broken horizontal line depicts the threshold value as calculated in equation (3) in Methods. In the top panels, the secondary structural elements are reported in correspondence to residue numbers: circles represent α-helical and squares represent β-sheet structures.

This calculation indicates that *Dpl *possesses a core of amino acids whose interactions concentrate a fraction of the stabilization energy for the protein (about 30% of the overall stabilization energy) much higher than that concentrated in the *PrP *nucleus (about 20%) [15]. The relative contribution of the stabilization core is calculated by determining the relative contribution to the total energy due to the interaction of residues corresponding to peaks over the threshold calculated using Eq. (2) in the Methods section (see ref. [15] for details). More interesting differences appear if one compares the variation of the energy spectrum of the first eigenvector for *Dpl *and *PrP *when the temperature is raised from 310 K to 350 K. In order to provide a quantitative estimate of the differences among the different profiles, we calculated the correlation coefficients between the corresponding components of the different eigenvectors. The results of the calculations are reported in Table 1. As noticed previously, the profile of the principal eigenvector of *PrP *at 350 K, pH 7 changes with respect to the situation at 310 K, their correlation coefficient being 0.71, and the distribution of peaks appears to become very similar to that of the first eigenvector for the trajectory calculated at 310 K or 350 K at pH 2 conditions, known to induce fibril formation *in vitro *(Fig. 5) (see [16] for the eigenvalue decomposition at low pH and ref. [18] for the experimental data). The main point here is that in all the cases where *PrP *is known to be in denaturing or misfolding conditions (high temperature at pH 7, or low pH), the profile of the principal eigenvector is characterized by the disappearance of the well defined stabilizing core of residues, with a general spreading of the stabilization energy along the whole sequence and a well defined difference with respect to the situation where the protein is known to be in native conditions (Fig. 5; Table 1).

The situation is different for *Dpl*. The profile of the principal eigenvector reporting the most stabilizing interactions in the native state is not changed when raising the temperature at pH7: the spectra of the first *Dpl *eigenvectors are almost superimposable at pH7 for the situations at 310 K and 350 K, their correlation coefficient being 0.93, a value consistently higher than that observed for the analogous *PrP *simulations. The different characteristics of the two main eigenvector profiles can be considered as an indication of the changes in the free-energy profiles of *Dpl *and *PrP *when raising the temperature from 310 to 350 K, differences which are highly dependent upon the sequences. As we noticed in ref. [15], in fact, the energy decomposition analysis described should be considered to yield an approximation of the free energy of the state around which the simulation is being carried out. The free energy landscape around one state of a protein can actually change with temperature, without substantial changes in conformational properties.

**Figure 6:**
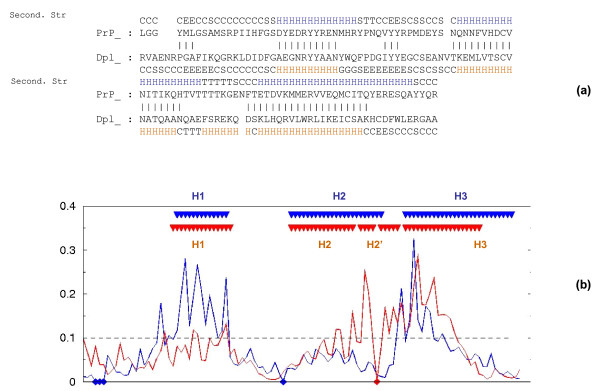
**Structural alignment and rescaling of the principal eigenvectors**. (a) Sequence alignment corresponding to the best structural alignment of the representatives of the most populated clusters for the simulations of PrP and Dpl at 310 K, obtained with the Sofist procedure. The RMSD of the aligned structures is 0.4 nm. (b) The components of the principal eigenvectors for PrP and Dpl rescaled and superimposed according to the optimal structural alignment.

**Figure 7:**
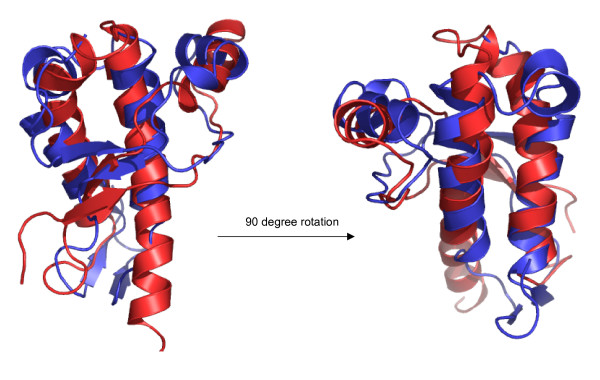
**3D representation of Structural Alignment**. 3D structural representation of the optimally aligned structures of PrP (red) and Dpl (blue).

This implies that the stabilization pattern of *Dpl *is conserved also in conditions which are known to trigger misfolding and aggregation in the far-related homologous *PrP*. In particular, the peaks with the highest intensity over the flat *t *value are always located in the H2' and H3 helices of *Dpl *and correspond to residues E120, Q132 and W136. To compare the role in protein stabilization of the physico-chemical properties of these residues with those on analogous secondary structure motifs on *PrP*, a structural alignment of the two proteins was necessary. The structural alignment was carried out on the representatives of the most populated structural clusters obtained from the two 50 ns simulations at 310 K, as described in Methods. This alignment strategy objectively identifies which substructures of *PrP *can be correlated with those of *Dpl*. After this operation is completed, the components of the principal eigenvector of each of the two proteins are compared simply by superimposing the peaks corresponding to residues belonging to the superimposed secondary structures in the aligned structures. This should allow an objective comparison of the profiles (Fig. 6a and 6b), independent of the simple sequence alignment which may be flawed by the low homology degree. As shown in Fig. 6b, the common part of the stabilizing cores is located in the N-terminal part of helix H3. A large difference can be noticed for the region of helix H1, showing high value components for the first eigenvector of *PrP *and low values for the corresponding first eigenvector of *Dpl*. These differences reflect the higher helical propensity of the sequence of *PrP *helix H1 compared to that of *Dpl*: helix H1 from the Prion protein was in fact observed to be structured in solution in isolation with NMR and other spectroscopic tools [19]. Moreover, Kuznetsov and Rackowsky [20] showed with comparative computational analysis that while several algorithms could correctly predict a helical conformation for *PrP *H1 sequence, this was not the case for the corresponding *Dpl *H1 sequence, which in most cases was predicted to have low helical propensity. This latter sequence, moreover, contains one chameleon sequence, which is not found in *PrP*. The other main significant difference is found in correspondence of helix H2' of *Dpl*, which is the part of the second helix following the kink, the only small but substantial structural difference between the two proteins (See Figs. 6 and 7). The comparison of the identities of residues corresponding to the highest peaks in *Dpl *with those in corresponding positions on *PrP *after the structural alignment shows that charged or highly polar residues in the former (E120, Q132) correspond to uncharged or hydrophobic residues on the latter (T190, V203), while the aromatic residue W136 on *Dpl *corresponds to a negatively charged moiety E207 on *PrP*, showing that the chemical properties of the residues belonging to the common cores are highly different. These three positions in the Prion Protein are all associated directly or are within one residue from sites whose mutations have been shown to induce disease or misfolding. Moreover, three residues conserved in *Dpl *and *PrP*, i.e. T113 (T183 in *PrP*), R133 (R208) and V184 (V210), which are part of the stabilizing core of *Dpl *are connected to genetic mutations related to increased probabilities of developing familial forms of TSE [21]. The presence of conserved residues, important for stability and implied in the onset of the misfolding diseases in one of two far related proteins, may be indicative of the common origin of a part of the folding nucleus. W136 in *Dpl*, in particular, is located at the center of the large hydrophobic cleft which differentiates the surfaces of *Dpl *and *Prp*. The role of the large Trp side chain in this case is to stabilize this particular hydrophobic surface. Luhrs and coworkers [21] noticed that the hydrophobic residues forming this surface are highly conserved in doppel proteins from different species, and might represent a binding site for unidentified, functionally important factors. Since this surface is absent in *PrP*, this view might also support a function that is unique to doppel proteins. A second important structural difference between the two proteins is the presence in *Dpl *of an additional S-S bridge between residues 94 and 143 connecting the loop between β2 and α2 with the C-terminal segment of the protein. This second S-S bridge is absent in *PrP*. Luhrs and coworkers showed [21] that this second S-S contributes to the aforementioned hydrophobic cleft on the one hand, while on the other it causes a rather dramatic structural difference when compared to the analogous region of *PrP*. Since this loop in *PrP *was suggested to be part of the "Protein X" recognition epitope, which seems to be involved in transmission and propagation of TSEs, this conformational difference was considered as a possible basis for the functional differences between the two proteins. Experimental NMR structural determinations on a double mutant of PrP with an additional disulfide bridge in the "protein X" binding site showed that the double mutation (M166C/E221C) could be accommodated with slight and strictly localized conformational changes [22]. The residues corresponding to the possible locations of the additional S-S bridge in PrP do not correspond to high peaks in the profile of the principal eigenvector (Fig. 5). This is consistent with the observations by Zahn *et al*. who showed that the insertion of a second disulfide bridge in the "protein X" epitope could be highly compatible with the structure of PrP.

It is interesting to observe at this point that also in *Dpl *the two Cys residues constituting the additional S-S bridge are not part of the folding nucleus of the protein. In this context, White and coworkers [23] noticed that while removal of the second disulphide bond from *Dpl *causes the melting temperature to decrease as expected from ~50°C to ~40°C, it does not affect the unfolding mechanism: no intermediate formation and no transition to β-rich structures is in fact observed. The fact that neither *Dpl *nor its mutant exhibited the α-β transformation typical of the prion protein suggest that this conversion property may actually be strictly dependent on the sequence differences in the folding nuclei of the two proteins.

Moreover, these observations show that similar topological organizations can be obtained by two significantly different sequences (25% homology) by a different distribution and organization of the stabilizing interactions. Strictly connected to the sequence-topology properties, the significant variation in the principal eigenvector profile upon temperature variation suggests possible different mechanisms for the unfolding/misfolding reactions of the two proteins. Interestingly, the most significant energy redistribution at 350 K characterizes *PrP*, which is known experimentally to undergo a transition to an intermediate structure.

Summing up, the results of the structural and energy decomposition analysis of the two proteins sharing the same topological organization show important differences in the stabilization mechanism of their native states and provide a possible rationale to explain the different unfolding-misfolding behaviors of the two molecules observed experimentally, which in turn has important pathological consequences. First of all, the nucleus of *Dpl *concentrates a much higher fraction of the global stabilization energy compared to *PrP*. Moreover, the distribution of stabilization energy in the former does not change with temperature, at variance with the latter.

These observations indicate that a different and more "solid" set of interactions have to be broken to unfold *Dpl*. In order to unfold *Dpl*, one has to break a network of interactions whose weight on stabilization is much higher than in the case of *PrP*. Once the more stable native interaction network of *Dpl *is broken, the protein can simply unfold to random coil or molten globule structures. The observation of Fig. 3 actually shows that the unfolding of *Dpl *is early and more cooperative than in the case of *PrP*, which undergoes a gradual unfolding without a single cooperative event. If one considers the reverse process, the folding of *Dpl *would require the formation of a more stable and extended folding nucleus, which would restrict the protein from exploring pathways alternative to the one leading to the native state, making this state energetically more accessible than alternative ones leading to different 3D structures.

**Figure 8:**
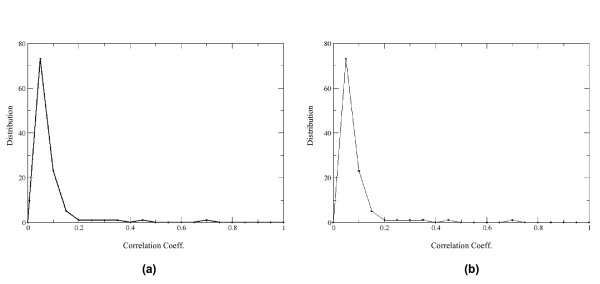
**310–450 K Correlation coefficient Superposition**. Distributions of the correlation coefficients of the superposition between the first eigenvector at 310 K and all the eigenvectors at 450 K for *PrP *(a) and *Dpl *(b).

*PrP*, whose network of stabilization contacts is looser than that for *Dpl*, can form different sets of interactions on the folding pathway and end up folding to a different energy minimum, the *PrP*^*Sc *^isoform. It is worth noting, at this point, that the profile of peaks for the first eigenvector after complete unfolding of *Dpl *at 450 K is totally different from the native one, in contrast to what happens for *PrP *(Fig. 5). In particular, the interaction spectrum for denatured *Dpl *does not show any particularly ordered patterns of interaction with intense peaks appearing all along the sequence. The correlation coefficient between the components of the principal eigenvectors at 310 and 450 K, 0.44, is significantly lower than that between the components of the principal eigenvectors at 310 and 350 K, 0.93. The *PrP *case is different: after 20 ns at 450 K, where the structure is denatured and rapidly refolded to a β-sheet rich conformation, the clusters of strong interactions are formed by the same residues as those present in the native state, although in a different 3D structural arrangement. In this case the correlation coefficient between the principal components for 310 and 450 K is 0.84, significantly higher than in the case of *Dpl*. This value for *PrP *significantly higher than the overlap between the principal components for 310 K and all the other eigenvectors at 450 K. In order to ensure that the above reported observations are not random, the superposition between the first eigenvector at 310 K and all the eigenvectors at 450 K were calculated and the distributions of the correlations coefficient are reported in Fig. 8a) and 8b). For both cases the distributions are highly peaked at values corresponding to correlation coefficients of 0.08 substantially showing that, except for the case first eigenvectors at 310 and 450 K of the PrP protein, no correlation is present between the components of the first eigenvector at 310 K and all the other eigenvectors at 450 K.

**Figure 9:**
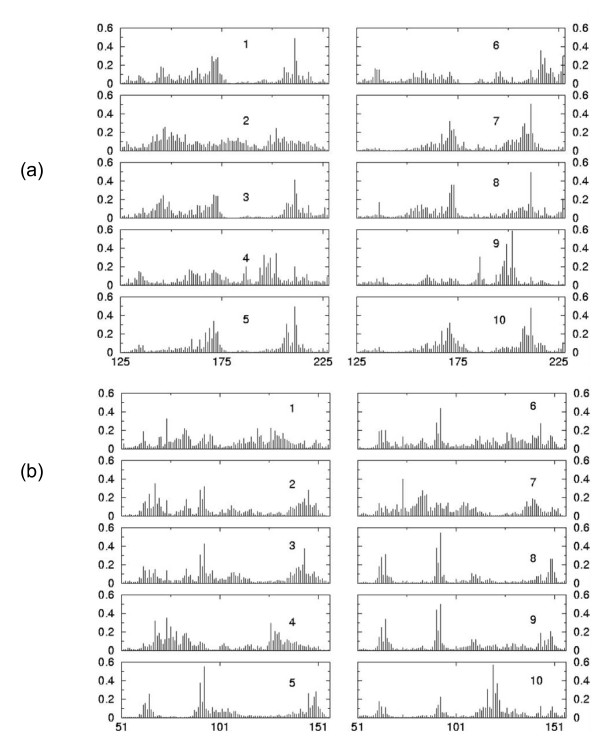
**Energy Decomposition for the 10 most populated conformational clusters at 450 K**. The components of the principal eigenvector of the residue-residue interaction matrices were calculated for the first 10 most populated conformational clusters for the simulations of *PrP *(a) and *Dpl *(b) at 450 K. The number in each graph corresponds to the cluster number.

**Figure 10:**
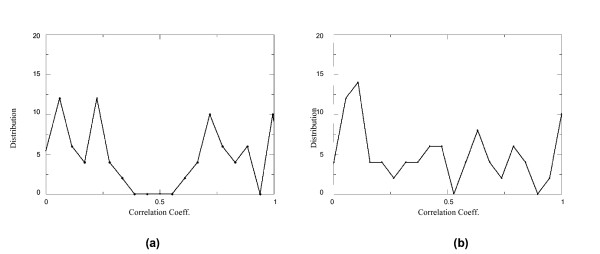
**Correlation coefficient distribution between all pairs of conformational clusters at 450 K**. The figure displays the distribution of correlation coefficients between all the pairs of conformational clusters in the simulations of *PrP *(a) and *Dpl *(b) at 450 K.

The picture that emerges from these results suggests that the *PrP *sequence can access multiple conformations all compatible with a similar energy distribution, while the *Dpl *sequence cannot. Clearly, at this stage of simulation and analysis, this model only represents a possible rationalization of experimental observations on two closely correlated proteins, and should not be considered as a diagnostic tool to predict in advance whether a certain sequence is able to misfold to different intermediates or not. A second important caveat that one should consider is that the set of conformations on which the analysis is carried out is highly heterogeneous at 450 K, where large conformational transitions occur and highly diverse sets of interactions may be present. To address this point, the components of the principal eigenvector of the residue-residue interaction matrices were calculated for the first 10 most populated conformational clusters for the simulations of *PrP *and *Dpl *at 450 K, Fig 9a and 9b. In the case of Prion the components of the first eigenvector calculated for each cluster are more correlated to one another and to the components of the first eigenvector of the native simulation. The distribution of correlation coefficients between all the pairs of eigenvectors belonging to different conformational clusters was also calculated (Fig 10a and 10b). The distribution for *PrP*, Fig 10a, displays two superclusters. Within each supercluster the eigenvectors are highly correlated (cf. the peak centered around 1), while pairs belonging to two different superclusters are not. The presence of these superclusters reflects the conformational variability actually present at 450 K. It is anyway interesting to note that this new set of conformations, despite being structurally dissimilar, displays remarkably similar energy distributions, thus being very different from a random collection of unfolded states. In the case of *Dpl*, in contrast, a sensitively higher degree of heterogeneity is observed also for the distribution of the components of the first eigenvector for each cluster (see Figs. 9b and 10b.). Clearly, in the case of high temperature simulations, and for *Dpl *in particular, one should be careful in drawing conclusions based on sets of highly heterogeneous conformations. However, the fact that in the case for *PrP *similar interaction profiles, superimposable to the native one, are conserved for a highly diverse set of conformations is suggestive of the chamaleontic properties of the prion sequence, able to access different states thorough a similar set of interactions.

These observations are consistent with the results obtained by other authors using a different approach to study the folding dynamics of *Dpl *and *PrP*. Settanni and coworkers, in particular, using a simplified potential biased towards the native topology, could show that while *Dpl *folds by crossing one main free energy barrier, *PrP *has two alternative folding pathways available [24].

Using a different approach, Fernandez and coworkers proposed a measure of amyloidogenic propensity relying on the analysis of the density of backbone hydrogen bonds exposed to water attack in monomeric structure [25]. On this basis, the authors proposed a diagnostic tool based on the identification of hydrogen bonds with a paucity of intramolecular dehydration or "wrapping", and used this predictor to successfully identify potentially pathogenic mutations that foster amyloidogenic propensity in human prions. When the same analysis was applied to *Dpl*, the wrapping measurements yielded a dramatically different level of amyloidogenic propensity. The authors suggested that that the packing within the fold, and not the fold itself, contains the signal for aggregation.

These observations are also consistent with what we observe herein. The higher number of stabilizing interactions in the nucleus of *Dpl *determine a tighter packing, and a lower tendency of water to disrupt intramolecular interactions favouring conformational transitions to the β-sheet rich structures characteristic of amyloids.

## Conclusion

The results of our comparative analysis of *PrP *and *Dpl*, sharing the same native topology despite a very low sequence homology, have provided valuable information on several aspects of the stabilization and (mis)-folding mechanisms of the two proteins. In particular, we could show that:

**1) **the stabilization core of *Dpl *provides a higher relative contribution to the overall stabilization energy compared to that of *PrP*.

**2) **the stabilization core of *Dpl *is stable and conserved also at 350 K. At this temperature, which is known to trigger misfolding and aggregation in *PrP*, the stabilization core of this latter protein is not conserved, and the whole stabilization energy is spread over the whole sequence favoring conformational interconversions to other structures.

**3) **As a consequence, *PrP *can misfold to different aggregation prone conformations, while *Dpl *cannot.

We think that our results are consistent and supportive of the experimental findings that Doppel lacks the scrapie isoform and that such remarkably different behavior is due to the presence of a different stabilization core, which in turn determines a different folding mechanism when compared to *PrP*.

From the practical point of view, we think that this type of analysis can be extended to other sequences which fold (or can be modeled) into the 3D structure typical of PrP as a relatively rapid diagnostic tool to predict mis-folding properties. This approach can also overcome the current limitations of all-atom MD simulations, which are still too computationally demanding to provide directly thermodynamical information about the folding and misfolding of a protein of the size of the two studied here. We have shown in fact that the shape of the principal eigenvector, which can be obtained with simulations accessible with present day computational power, can clearly distinguish the conditions which promote misfolding from those which do not.

## Methods

### Structures, simulation set-up and analysis

The starting structures for the all-atom MD simulations of the Doppel Protein (*Dpl*, fragment 51–157) and for the human Prion Protein (*PrP*, fragment 125–229) were taken from the protein data bank, with codes 1I17.pdb [13] and 1QLZ.pdb [14].

To mimic the solution conditions at pH 7, Lysin amino groups were considered protonated, while the carboxyl groups were considered to bear a negative charge. In the case of *Dpl*, the total formal charge on the protein resulted to be +1 and one Chloride counterion was added to ensure electroneutrality of the simulation box; in the case of *PrP *The total charge on the protein was -3 and three Sodium ions were added to ensure electroneutrality of the system.

The proteins were solvated with water in a octahedral box large enough to contain 1.2 *nm *of solvent around the peptide. The simple point charge (SPC) water model was used [26] to solvate each protein in the simulation box. Each system was subsequently energy minimized with a steepest descent method for 1000 steps. The calculation of electrostatic forces utilized the PME implementation of the Ewald summation method. The LINCS [27] algorithm was used to constrain all bond lengths. For the water molecules the SETTLE algorithm [28] was used. A dielectric permittivity, ε = 1, and a time step of 2 fs were used. All atoms were given an initial velocity obtained from a Maxwellian distribution at the desired initial temperature of 300 K. The density of the system was adjusted performing the first equilibration runs at NPT condition by weak coupling to a bath of constant pressure (P_0 _= 1 bar, coupling time τ_*P *_= 0.5 ps) [29]. In all simulations the temperature was maintained close to the intended values by weak coupling to an external temperature bath [29] with a coupling constant of 0.1 ps. The peptide and the rest of the system were coupled separately to the temperature bath.

In both cases, the protein was simulated at 310 K for 50 ns, then the temperature was raised at 350 K for the next 20 ns and finally, after this period, each system was heated up to 450 K for 20 more ns, resulting in a total simulation time of 90 ns for each of the two studied systems. All simulations were run at NPT conditions.

All simulations and analysis were carried out using the GROMACS package (version 3.2) [30-32], using the GROMOS96 43A1 force field [33]. All calculations were performed on clusters of PCs, with Linux operating system. Graphical display of structures was done using the PyMOL software. Structural alignments were carried out with the Sofist algorithm [34] on the representatives of the most populated clusters for the 50 ns 310 K simulations. Structural Clusters were defined using the structural clustering algorithm proposed by Daura and coworkers [35].

### Energy decomposition analysis

The basic idea behind the energy decomposition analysis is to extract energetic information on the protein from molecular dynamics (MD) simulations, and from it to gain insight into the determinants of the stability of the native protein conformation, and their influence on the folding process [15,36]. The main information needed to achieve this goal is the interaction matrix *M*_*ij*_, calculated averaging the corresponding interaction energies, comprising all the non-bonded inter-residue energy components (e.g. van der Waals and Electrostatic), over a MD trajectory starting from the native conformation. The matrix *M*_*ij *_can be decomposed in eigenvalues, in the form

Mij=∑α=1Nλαμiαμjα,     (1)
 MathType@MTEF@5@5@+=feaafiart1ev1aaatCvAUfKttLearuWrP9MDH5MBPbIqV92AaeXatLxBI9gBaebbnrfifHhDYfgasaacH8akY=wiFfYdH8Gipec8Eeeu0xXdbba9frFj0=OqFfea0dXdd9vqai=hGuQ8kuc9pgc9s8qqaq=dirpe0xb9q8qiLsFr0=vr0=vr0dc8meaabaqaciaacaGaaeqabaqabeGadaaakeaacqWGnbqtdaWgaaWcbaGaemyAaKMaemOAaOgabeaakiabg2da9maaqahabaGaeq4UdW2aaSbaaSqaaiabeg7aHbqabaaabaGaeqySdeMaeyypa0JaeGymaedabaGaemOta4eaniabggHiLdGccqaH8oqBdaqhaaWcbaGaemyAaKgabaGaeqySdegaaOGaeqiVd02aa0baaSqaaiabdQgaQbqaaiabeg7aHbaakiabcYcaSiaaxMaacaWLjaWaaeWaceaacqaIXaqmaiaawIcacaGLPaaaaaa@4AAC@

where *N *is the number of amino acids in the protein, λ_α _is an eigenvalue and μiα
 MathType@MTEF@5@5@+=feaafiart1ev1aaatCvAUfKttLearuWrP9MDH5MBPbIqV92AaeXatLxBI9gBaebbnrfifHhDYfgasaacH8akY=wiFfYdH8Gipec8Eeeu0xXdbba9frFj0=OqFfea0dXdd9vqai=hGuQ8kuc9pgc9s8qqaq=dirpe0xb9q8qiLsFr0=vr0=vr0dc8meaabaqaciaacaGaaeqabaqabeGadaaakeaacqaH8oqBdaqhaaWcbaGaemyAaKgabaGaeqySdegaaaaa@3189@ are the components of the associated eigenvector. We assume that the eigenvectors are normalized to unity and, since *M*_*ij *_is symmetrical, all the eigenvalues are real.

For the sake of simplicity, we label the *N *eigenvalues in increasing order, so that λ_1 _is the most negative. Accordingly, the different terms in the sum in Eq. (1) approximate the real interaction energy *M*_*ij *_to an increasing extent, the first term containing the largest contribution to the stabilization of the native conformation. The components μi1
 MathType@MTEF@5@5@+=feaafiart1ev1aaatCvAUfKttLearuWrP9MDH5MBPbIqV92AaeXatLxBI9gBaebbnrfifHhDYfgasaacH8akY=wiFfYdH8Gipec8Eeeu0xXdbba9frFj0=OqFfea0dXdd9vqai=hGuQ8kuc9pgc9s8qqaq=dirpe0xb9q8qiLsFr0=vr0=vr0dc8meaabaqaciaacaGaaeqabaqabeGadaaakeaacqaH8oqBdaqhaaWcbaGaemyAaKgabaGaeGymaedaaaaa@30DA@ of the associated eigenvector indicate to which extent each amino acid participates to the stabilization. In other words, each term in Eq. (1) accounts for an amount of energy λ_α _which is shared among the different residues according to the corresponding eigenvector μiα
 MathType@MTEF@5@5@+=feaafiart1ev1aaatCvAUfKttLearuWrP9MDH5MBPbIqV92AaeXatLxBI9gBaebbnrfifHhDYfgasaacH8akY=wiFfYdH8Gipec8Eeeu0xXdbba9frFj0=OqFfea0dXdd9vqai=hGuQ8kuc9pgc9s8qqaq=dirpe0xb9q8qiLsFr0=vr0=vr0dc8meaabaqaciaacaGaaeqabaqabeGadaaakeaacqaH8oqBdaqhaaWcbaGaemyAaKgabaGaeqySdegaaaaa@3189@

If the second eigenvalue λ_2 _is much higher than λ_1_, one can approximate the whole interaction matrix as

Mij≈λ1μi1μj1,     (2)
 MathType@MTEF@5@5@+=feaafiart1ev1aaatCvAUfKttLearuWrP9MDH5MBPbIqV92AaeXatLxBI9gBamXvP5wqSXMqHnxAJn0BKvguHDwzZbqegyvzYrwyUfgarqqtubsr4rNCHbGeaGqiA8vkIkVAFgIELiFeLkFeLk=iY=Hhbbf9v8qqaqFr0xc9pk0xbba9q8WqFfeaY=biLkVcLq=JHqVepeea0=as0db9vqpepesP0xe9Fve9Fve9GapdbaqaaeGacaGaaiaabeqaamqadiabaaGcbaGaemyta00aaSbaaSqaaiabdMgaPjabdQgaQbqabaGccqGHijYUcqaH7oaBdaWgaaWcbaGaeGymaedabeaakiabeY7aTnaaDaaaleaacqWGPbqAaeaacqaIXaqmaaGccqaH8oqBdaqhaaWcbaGaemOAaOgabaGaeGymaedaaOGaeiilaWIaaCzcaiaaxMaadaqadiqaaiabikdaYaGaayjkaiaawMcaaaaa@529A@

reducing the information needed to specify the interaction from *N*^2 ^to *N *numbers.

The network of interactions containing most of the information on the stabilization energy is then determined by analyzing the first eigenvector and identifying those sites whose component is higher than a threshold value *t*. This is calculated as the value corresponding to a normalized vector whose components provide the same contribution for each site (flat eigenvector). This corresponds, to a first approximation, to a situation in which each residue contributes with the same weight to structural stability. In this approximation the threshold value depends only on the number *N *of residues in the protein and is calculated as:

t=1N     (3)
 MathType@MTEF@5@5@+=feaafiart1ev1aaatCvAUfKttLearuWrP9MDH5MBPbIqV92AaeXatLxBI9gBaebbnrfifHhDYfgasaacH8akY=wiFfYdH8Gipec8Eeeu0xXdbba9frFj0=OqFfea0dXdd9vqai=hGuQ8kuc9pgc9s8qqaq=dirpe0xb9q8qiLsFr0=vr0=vr0dc8meaabaqaciaacaGaaeqabaqabeGadaaakeaacqWG0baDcqGH9aqpdaGcaaqaamaalaaabaGaeGymaedabaGaemOta4eaaaWcbeaakiaaxMaacaWLjaWaaeWaceaacqaIZaWmaiaawIcacaGLPaaaaaa@3530@

In the case of *Dpl *the value of *t *is 0.097, while in the case of *PrP *this value equals 0.098.

## Authors' contributions

SC carried out the molecular dynamics studies and analysis and developed analysis software and drafted a first version of the manuscript. GT participated in the design of the study and in the development of the energy analysis method. GC conceived of the study, participated in its design and coordination and wrote the manuscript.

## Supplementary Material

Additional File 1

## Figures and Tables

**Table 1:** Correlation coefficients between and component values of the principal eigenvectors for *PrP *and * Dpl *at different temperatures

	*PrP *350 K	*PrP *450 K	*Dpl *350 K	*Dpl *450 K
*PrP *310 K	0.71	0.84		
*PrP *350 K		0.48		
*Dpl *310 K			0.93	0.44
*Dpl *350 K				0.57
