# Supplementary material for: Similar folds with different stabilization mechanisms: the cases of prion and doppel proteins
Source: BMC Struct Biol. 2006 Jul 21;6:17. doi: 10.1186/1472-6807-6-17 (PMC1574322; doi:10.1186/1472-6807-6-17)
Supplement: Additional File 1 [file 1472-6807-6-17-S1.PPT]

## Slide 1
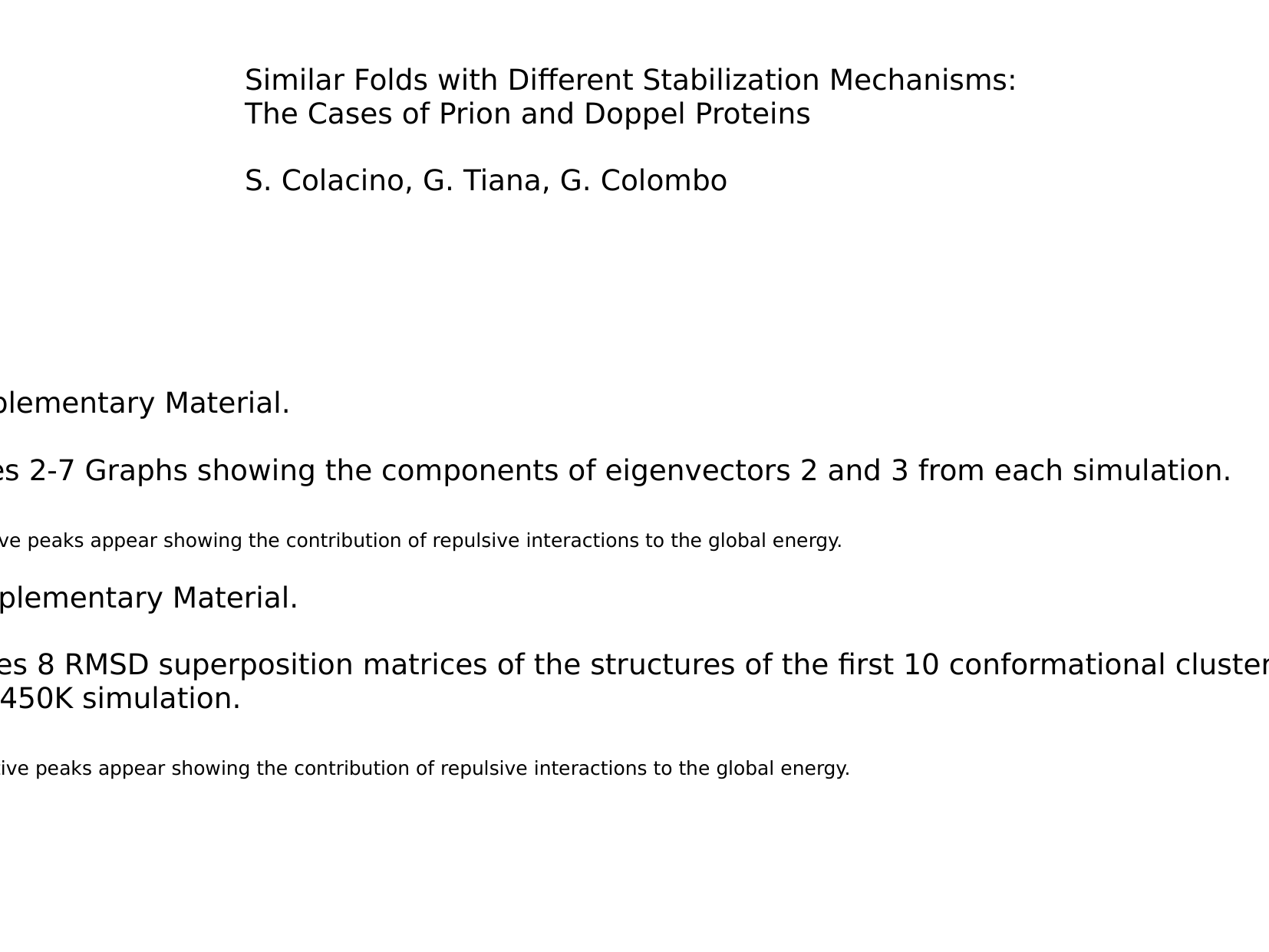

Similar Folds with Different Stabilization Mechanisms: The Cases of Prion and Doppel Proteins
S. Colacino, G. Tiana, G. Colombo
Supplementary Material.
Pages 2-7 Graphs showing the components of eigenvectors 2 and 3 from each simulation.
Negative peaks appear showing the contribution of repulsive interactions to the global energy.
Supplementary Material.
Pages 8 RMSD superposition matrices of the structures of the first 10 conformational clusters from
the 450K simulation.
Negative peaks appear showing the contribution of repulsive interactions to the global energy.

## Slide 2
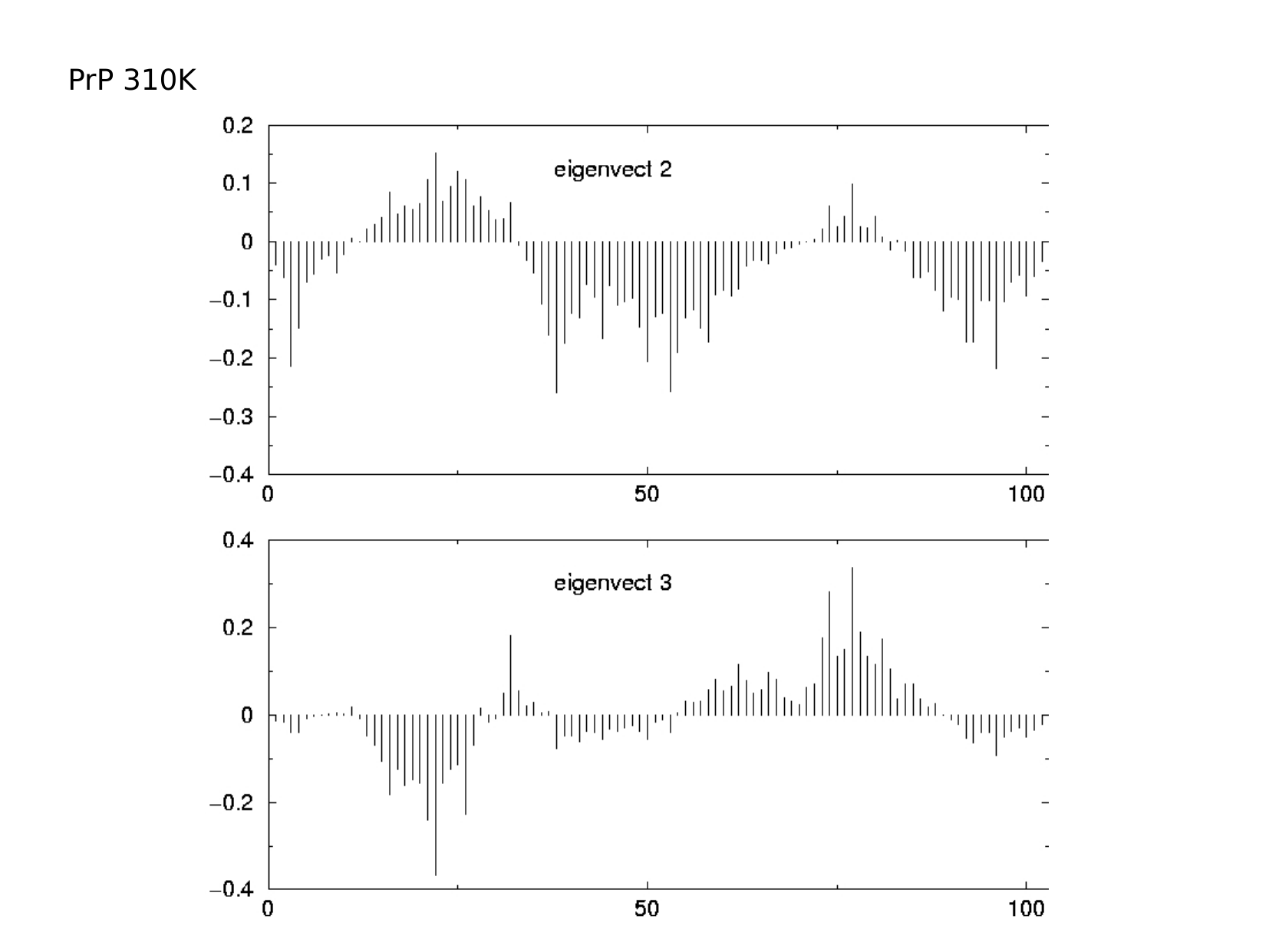

PrP 310K

## Slide 3
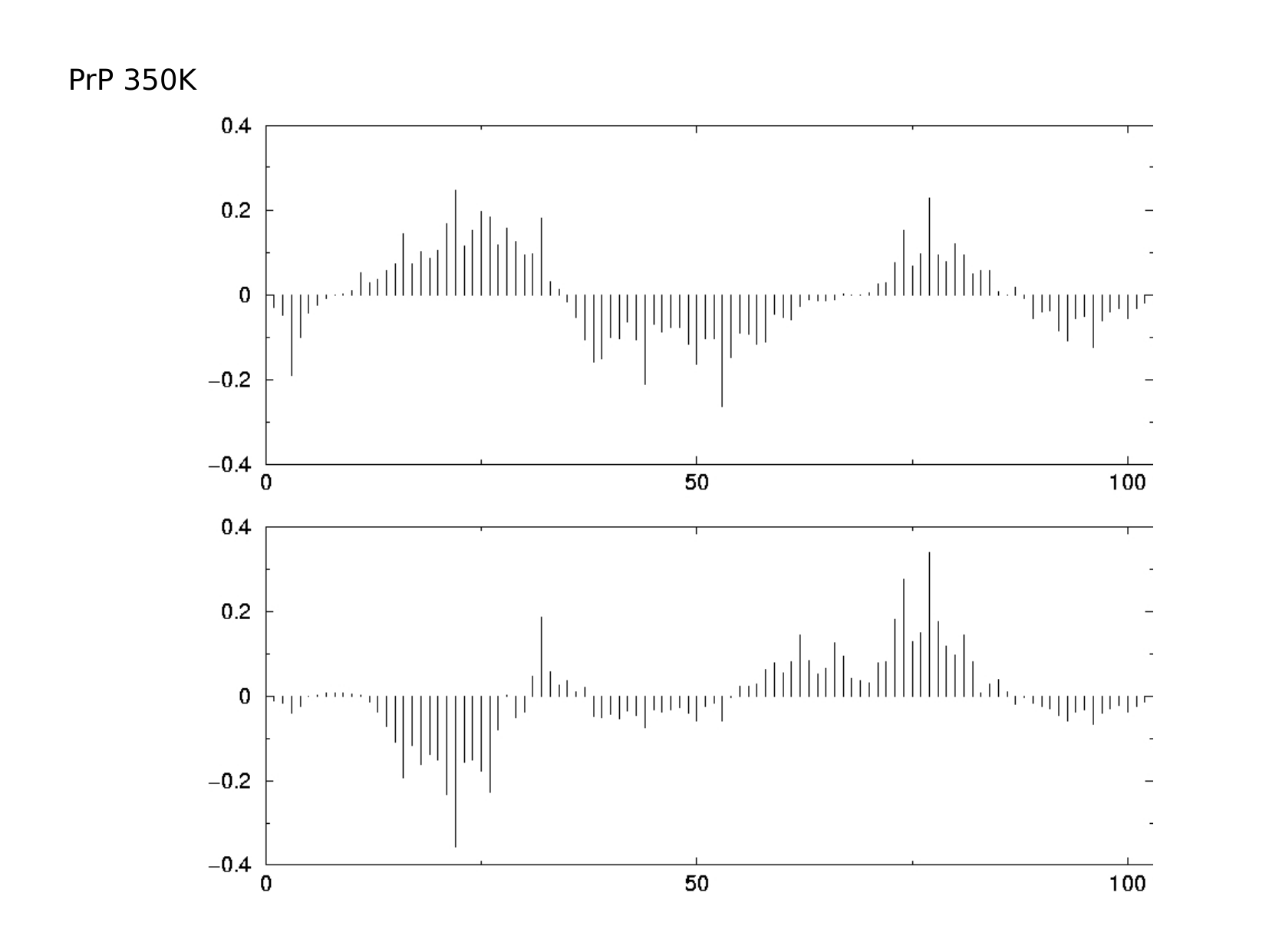

PrP 350K

## Slide 4
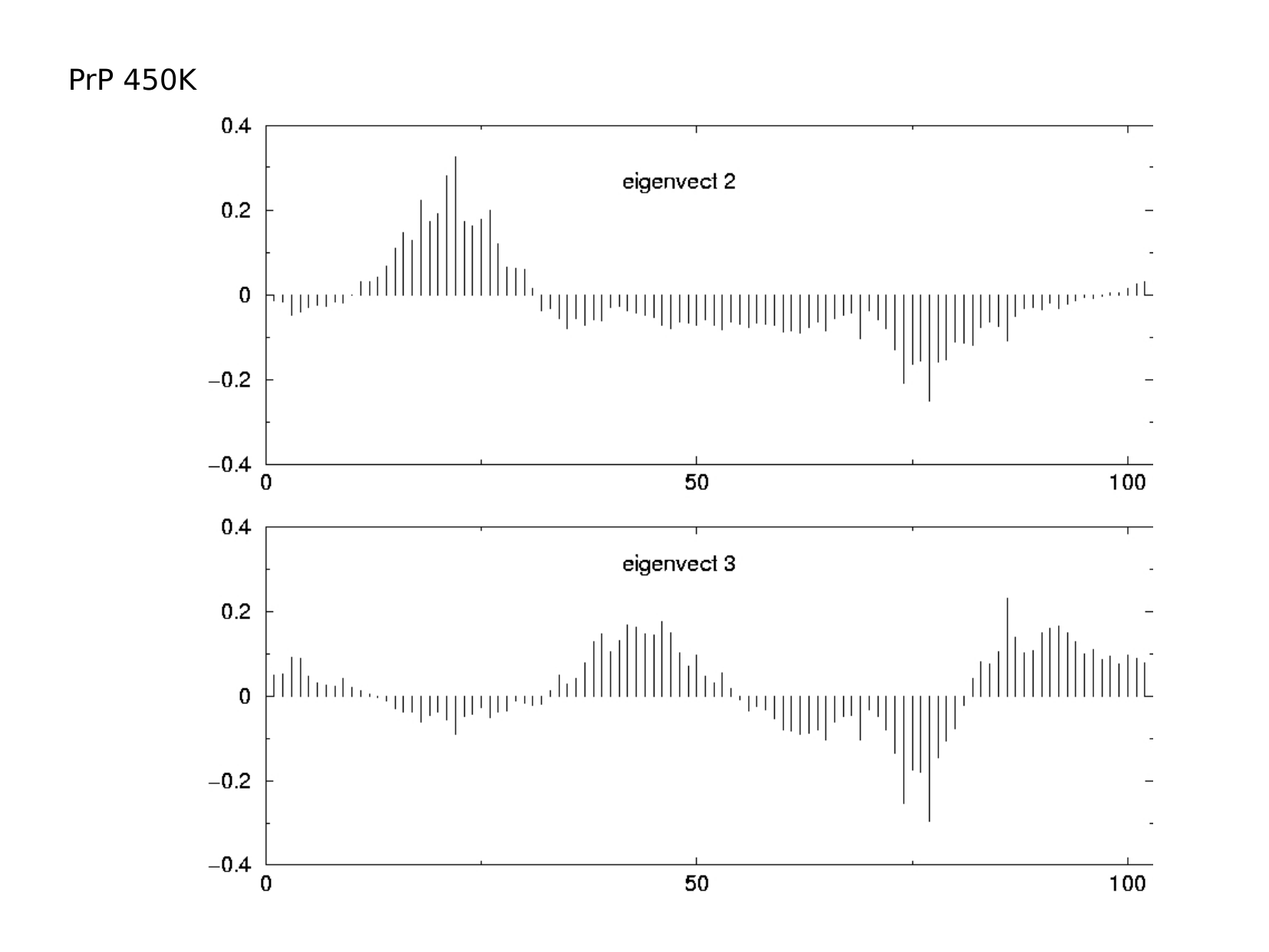

PrP 450K

## Slide 5
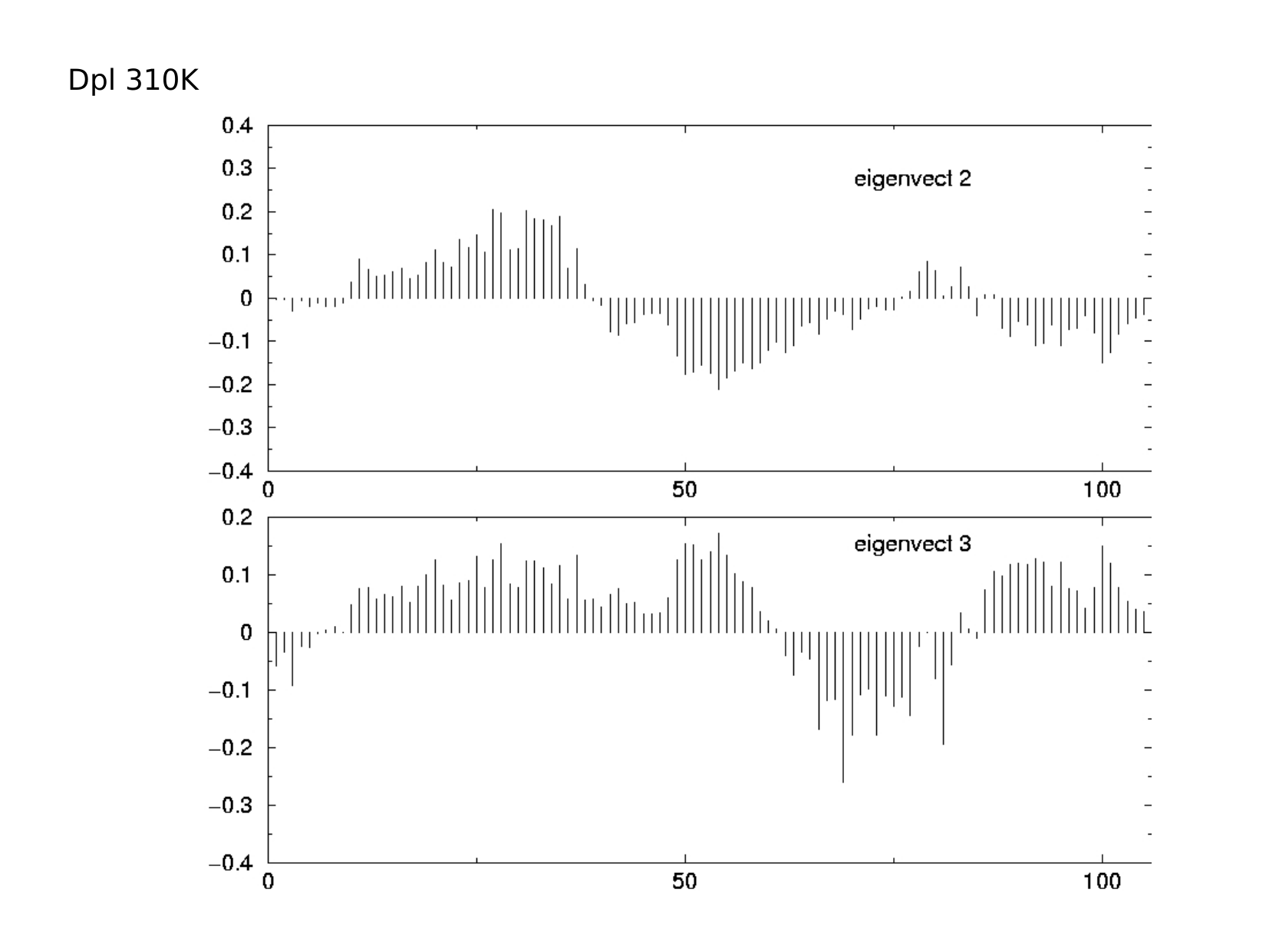

Dpl 310K

## Slide 6
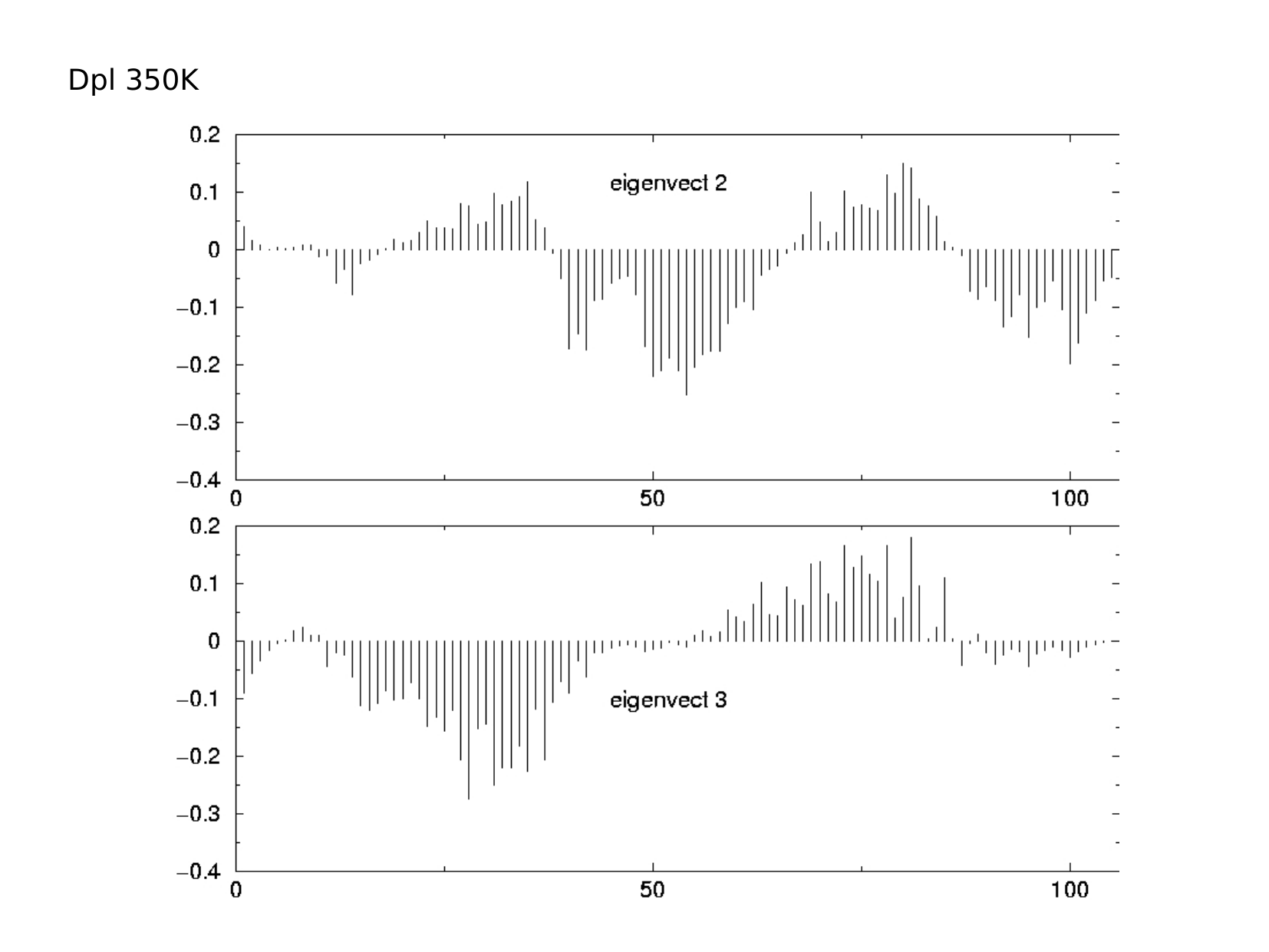

Dpl 350K

## Slide 7
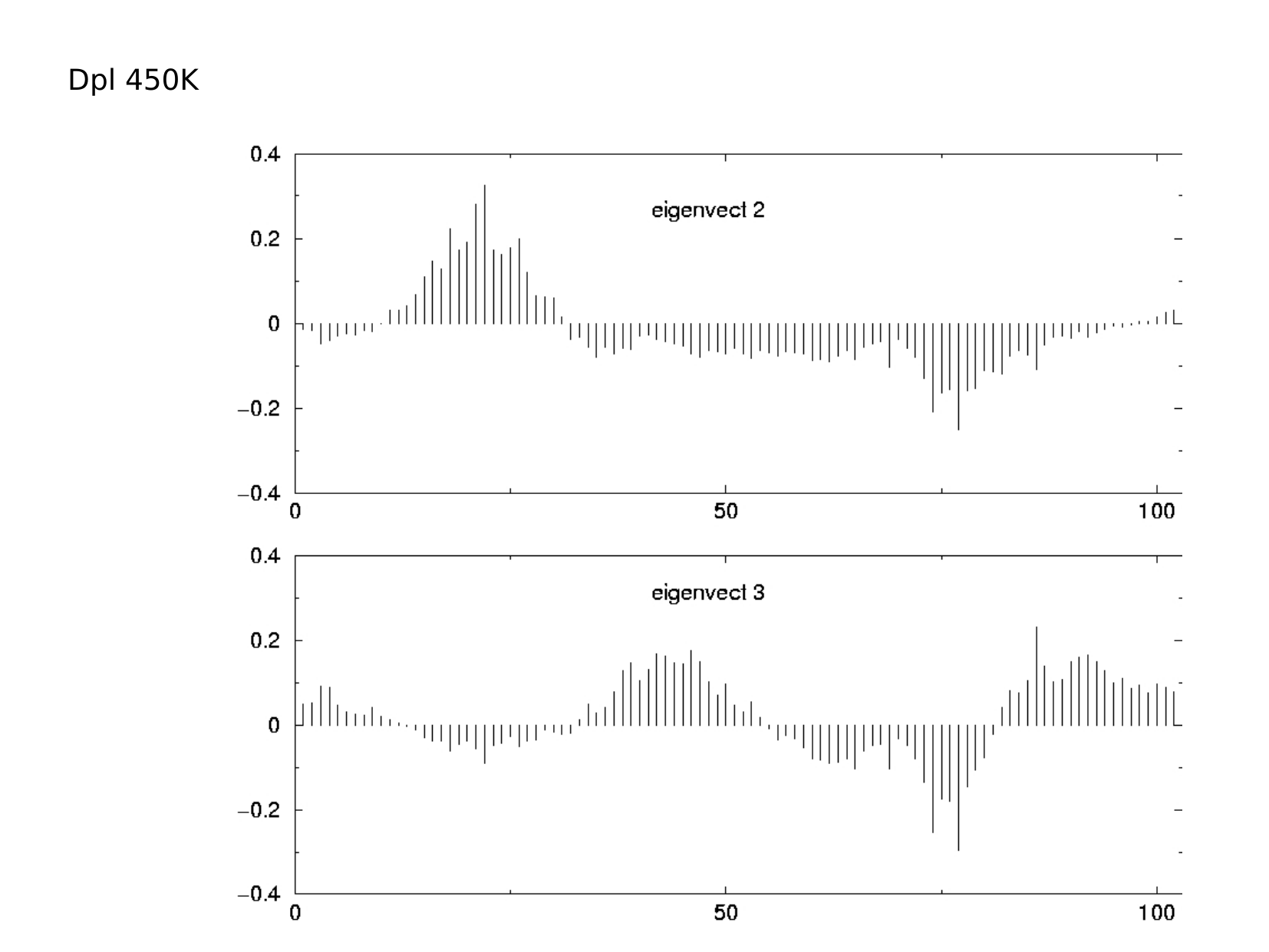

Dpl 450K

## Slide 8
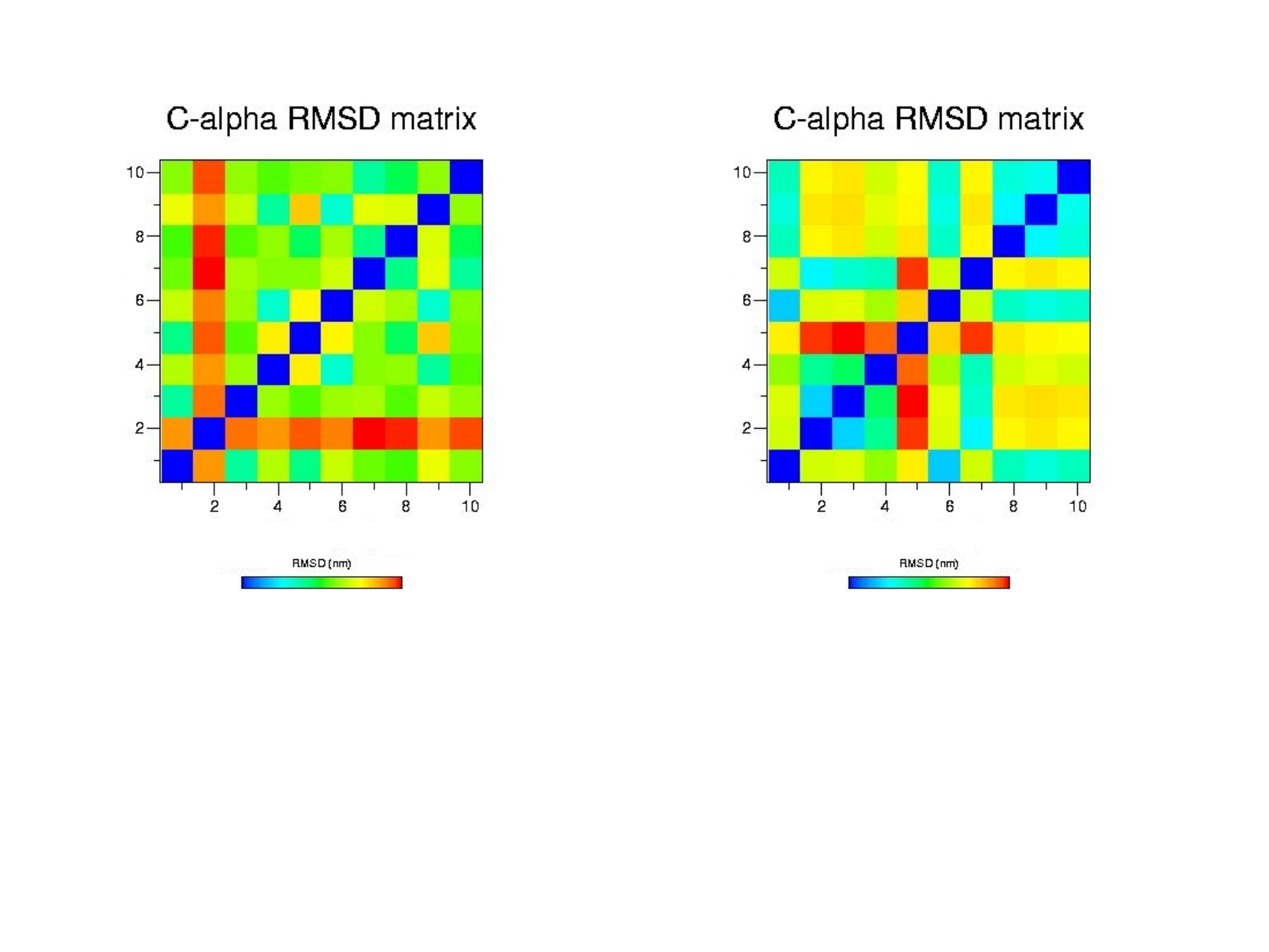

Prion
Doppel
Cluster number
Cluster number
Cluster number
Cluster number
0
1.4
0
1.4
